# Meteorological Observations, August

**Published:** 1860-10

**Authors:** J. G. Westmoreland


### METEOROLOGICAL OBSERVATIONS,

Taken by J. G. Westmoreland, M. D., for the Smithsonian Institution, August, 1860, at Atlanta, Ga., Latitude 33 deg. 45 min.—Longitude 7 deg. 30 min.—Height above the Sea, 1050 feet.

| Days of Month | Barometer. | | | Thermometer. | | | Rain and Snow. | | Clouds. | | | Wind. | | |
|---|---|---|---|---|---|---|---|---|---|---|---|---|---|---|
| | 7 A.M. | 2 P.M. | 9 P.M. | 7 A.M. | 2 P.M. | 9 P.M. | Amt. Rn | In. Sn'w | 7 A.M. | 2 P.M. | 9 P.M. | 7 A.M. | 2 P.M. | 9 P.M. |
| 1 | 30.05 | 30.15 | 30.15 | 74 | 78 | 76 | | | 0 | 0 | 0 | W | N W | W |
| 2 | 30.15 | 30.20 | 30.20 | 70 | 85 | 89 | | | 0 | 0 | 0 | W | W | W |
| 3 | 30.20 | 30.25 | 30.20 | 74 | 80 | 76 | | | 0 | 0 | 0 | W | W | W |
| 4 | 30.20 | 30.20 | 30.20 | 70 | 78 | 76 | | | 0 | 0 | 0 | W | W | W |
| 5 | 30.15 | 30.20 | 30.15 | 76 | 90 | 80 | | | 5 | 0 | 5 | W | N E | N E |
| 6 | 30.15 | 30.15 | 30.10 | 78 | 90 | 80 | | | 0 | 0 | 0 | W | N E | N E |
| 7 | 30.10 | 30.15 | 30.10 | 76 | 92 | 80 | | | 0 | 5 | 5 | N E | N E | N E |
| 8 | 30.10 | 30.10 | 30.00 | 80 | 94 | 82 | | | 0 | 0 | 0 | N E | N W | W |
| 9 | 29.95 | 30.05 | 30.00 | 76 | 72 | 80 | .37 | | 5 | 0 | 10 | S W | N E | S W |
| 10 | 30.00 | 30.05 | 30.00 | 74 | 90 | 80 | | | 10 | 5 | 10 | W | W | E |
| 11 | 29.95 | 30.00 | 30.00 | 70 | 68 | 70 | 1.50 | | 10 | 10 | 10 | E | E | E |
| 12 | 30.00 | 29.90 | 29.80 | 68 | 70 | 66 | | | 10 | 10 | 10 | E | E | E |
| 13 | 29.75 | 29.95 | 29.95 | 62 | 70 | 66 | 2.50 | | 10 | 10 | 5 | E | N E | N W |
| 14 | 29.95 | 30.05 | 30.15 | 64 | 76 | 66 | | | 0 | 5 | 0 | N W | N W | N |
| 15 | 30.10 | 30.15 | 30.15 | 62 | 76 | 68 | | | 0 | 0 | 0 | E | E | E |
| 16 | 30.20 | 30.20 | 30.20 | 62 | 72 | 68 | | | 0 | 0 | 0 | E | E | E |
| 17 | 30.20 | 30.20 | 30.15 | 66 | 82 | 78 | | | 0 | 0 | 0 | E | E | E |
| 18 | 30.10 | 30.15 | 30.10 | 70 | 88 | 80 | | | 0 | 0 | 0 | N E | W | W |
| 19 | 30.10 | 30.15 | 30.05 | 78 | 90 | 68 | .25 | | 5 | 0 | 10 | W | N W | N W |
| 20 | 30.05 | 30.15 | 30.10 | 66 | 84 | 74 | | | 5 | 0 | 0 | W | W | W |
| 21 | 30.10 | 30.10 | 30.00 | 70 | 84 | 70 | .75 | | 5 | 5 | 10 | W | N W | N |
| 22 | 30.00 | 30.10 | 30.05 | 70 | 82 | 74 | | | 5 | 0 | 10 | N W | N | N |
| 23 | 30.00 | 30.15 | 30.00 | 70 | 86 | 76 | | | 0 | 0 | 10 | W | W | W |
| 24 | 30.00 | 30.00 | 30.00 | 70 | 84 | 70 | .25 | | 10 | 0 | 10 | W | W | W |
| 25 | 29.95 | 30.05 | 30.00 | 68 | 82 | 70 | | | 10 | 0 | 0 | W | W | W |
| 26 | 30.00 | 30.10 | 30.05 | 68 | 84 | 70 | | | 0 | 0 | 5 | W | N W | W |
| 27 | 30.00 | 30.12 | 30.10 | 70 | 82 | 74 | | | 5 | 5 | 0 | W | W | W |
| 28 | 30.10 | 30.05 | 30.05 | 72 | 72 | 78 | 1.75 | | 5 | 10 | 10 | S W | S W | S W |
| 29 | 30.05 | 30.10 | 30.05 | 70 | 80 | 78 | .25 | | 10 | 5 | 5 | S W | W | W |
| 30 | 30.05 | 30.10 | 30.15 | 68 | 78 | 70 | | | 5 | 5 | 0 | W | W | W |
| 31 | 30.00 | 30.00 | 29.95 | 70 | 84 | 78 | | | 0 | 0 | 0 | W | W | W |

Explanation of Table.—0 Signifies Entire Clearness—5 Half Cloudy—10 Entire Cloudiness.

